# Chronic Traumatic Encephalopathy in Professional American Football Players: Where Are We Now?

**DOI:** 10.3389/fneur.2018.00445

**Published:** 2018-06-19

**Authors:** Tharmegan Tharmaratnam, Mina A. Iskandar, Tyler C. Tabobondung, Iqdam Tobbia, Prasaanthan Gopee-Ramanan, Taylor A. Tabobondung

**Affiliations:** ^1^School of Medicine, Royal College of Surgeons in Ireland, Dublin, Ireland; ^2^School of Medicine, Royal College of Surgeons in Ireland-Bahrain, Al Muharraq, Bahrain; ^3^Department of Family Medicine, Michael G. DeGroote School of Medicine, McMaster University, Brantford General Hospital, Hamilton, ON, Canada; ^4^Department of Pathology and Clinical Microbiology, School of Medicine, Royal College of Surgeons in Ireland-Bahrain, Adliya, Bahrain; ^5^Hamilton Health Sciences Centre, Department of Radiology, Michael G. DeGroote School of Medicine, McMaster University, Hamilton, ON, Canada; ^6^Department of Physical and Environmental Sciences, University of Toronto, Toronto, ON, Canada

**Keywords:** chronic traumatic encephalopathy, American football, traumatic brain injury, tau, neurodegeneration, tau protein, neurofibrillary tangles

## Abstract

Repetitive head trauma provides a favorable milieu for the onset of inflammatory and neurodegenerative processes. The result of long-lasting head trauma is chronic traumatic encephalopathy (CTE), a disease process well-recognized in boxers, military personnel, and more recently, in American football players. CTE is a chronic neurodegenerative disease with hallmarks of hyperphosphorylated tau (p-tau) aggregates and intercellular lesions of neurofibrillary tangles. The criteria for CTE diagnosis requires at least 1–2 focal perivascular lesions of p-tau in the cerebral cortex, at the depth of the sulci. These pathognomonic lesions aggregate within neurons and glial cells such as astrocytes, and cell processes within the vicinity of small blood vessels. CTE presents in a distinct topographical distribution pattern compared to other tauopathies such as AD and other age-related astrogliopathies. CTE also has an insidious onset, years after repetitive head trauma. The disease course of CTE is characterized by cognitive dysfunction, behavioral changes, and can progress to altered motor function with parkinsonian-like manifestations in later stages. This short review aims to summarize CTE in professional football, epidemiology, diagnosis based on neuroanatomical abnormalities, cognitive degeneration, and adverse mental health effects, as well as gaps in the literature and future directions in diagnostics, therapeutics, and preventive measures.

## Introduction

Insight into repetitive brain trauma was first demonstrated by Martland in 1928, who characterized boxers to display “punch-drunk syndrome” from repeated blows to the head (1). Later on, this phenomenon was coined as *dementia pugilistica*: a term used to describe a physical-psychic syndrome that accumulated over a lengthy boxing career, which was less begrudged amongst boxers than “punch drunk” (2). Today this is referred to as chronic traumatic encephalopathy (CTE). CTE is defined as brain trauma that is either repetitive, episodic, or a single precipitating event, leading to progressive brain neurodegeneration (3). The exact pathophysiology between concussion and CTE is yet to be definitively explained, but is believed to be due to axonal perturbations as a consequence of brain trauma. This causes metabolic abnormalities and cytoskeletal disturbances in the neuronal milieu such as microtubule destabilization, leading to disease propagation via hyperphosphorylated p-tau accumulation (4). This finding has been well documented in athletes involved in contact sports such as American football and boxing, but also in non-civilian populations such as the military (5, 6). Recent studies have highlighted the prevalence of neurotrauma and CTE in American football (NFL) players. Omalu and colleagues were the first to demonstrate this phenomenon in 2005, through a series of case-reports based on autopsy findings of deceased players brains (7–10). Others have shown the impacts of repeated neurotrauma to manifest in cognitive impairment, neuroimaging anomalies (11–13), and disruption in the cytological architecture of the brain (14–16). Considering the large number of athletes engaged in contact sports such as football, this is a noteworthy public health concern that merits public attention and interests clinicians and scientists alike. For the purposes of this mini review, our discussion will be restricted to American football. Herein, we provide a primer on risk factors for CTE in NFL players, the distinguishing characteristics of this neurodegenerative condition, pathophysiology, and its association with suicide.

## Mechanisms of injury in the development of CTE

American Football involves repetitive head trauma causing the brain to experience acceleration and deceleration forces (14, 17, 18), leading to coup/countercoup injuries. This can manifest in varying severity from concussions, or concussions of lesser magnitude (sub-concussion) to loss of consciousness (19). Over time, improperly managed brain trauma with inadequate recovery periods allow chronic inflammatory processes to lay the framework for CTE (14). Indeed, brain trauma without concussion can also lead to CTE (20, 21). It is believed that repetitive brain trauma leads to CTE through tau oligomerization following axonal deformation and microtubular destabilization (22). Over time, tau oligomers develop into paired helical, and straight filamentous neurofibrillary tangles (NFTs) (10, 13), which interfere with white matter tracts in the brain and cause signaling and communication abnormalities through denervation injury (23–25). This aggregation of NFTs leads to tau propagation in a prion-like manner (26). Overall, there is an inflammatory state through cytokine and chemokine activation which can lead to an increased blood-brain barrier permeability (27). Omalu suggests that brain trauma results in kinase accumulation from neuroinflammatory processes (3) such as free radical generation from oxidative stress, microglia upregulation, mitochondrial dysfunction, and calcium imbalance, which result in hyperphosphorylated tau (3, 26, 28, 29) Recent work has also highlighted the role of tau acetylation (ac-tau) in the progression of CTE, and increased major histocompatibility receptor expression on neurons (27, 30). In lieu of these findings, the exact mechanism by which tau hyperphosphorylation leads to CTE from brain trauma remains yet to be definitively explained (3, 21).

## Risk factors for CTE

Current literature suggests that the primary risk factor for the development of CTE is repetitive head trauma (5, 18, 31–34). Previous work by Guskiewicz et al. (35) has shown 61% of NFL players to have experienced at least 1 concussion within their careers (35). On the other hand, multiple concussions have been shown to be a risk factor for cognitive neurodegeneration and adverse effects on mental health in some individuals (35, 36), which may predispose them to develop CTE later in life. Generally, recovery can be expected from a single head impact with no CTE (19), however, experiencing 3 or more concussions is correlated to an increased risk of prolonged symptoms (37, 38). However, although documented cases of CTE are characterized by repetitive brain trauma, not all athletes exposed to brain trauma will necessarily develop CTE (39). Additionally, the frequency, duration, and magnitude of brain injury necessary to elicit the manifestations of CTE are not well understood in the literature (17, 19).

Genetic susceptibility has also been shown to be a predisposing factor in CTE development. Apolipoprotein (Apoε) is a 34-kDa glycosylated protein implicated as the primary source of cholesterol transport in the brain for neuronal repair (40). Individuals possessing the Apoε4 allele have been shown to have an increased risk of poor functional outcome (19). Compared to other isoforms, the Apoε4 variant has been associated with longer recovery times from neurotrauma, increased injury severity, and greater cognitive deficits in football players (41–43). Kutner et al. (43) also suggest that football players with the Apoε4 allele experience worse clinical outcomes, compared to carriers of other isoforms (43). In a study by McKee et al. (14), 50% of CTE cases were shown to carry one ε4 allele, with one case demonstrating homozygosity (14). The Apoε4 allele has also been associated with β-amyloid deposition in CTE. However, more recent work has emerged to show that Apoε variation in CTE cases is no greater than that of the general population (15, 33).

In ex-NFL players, age of first exposure (AFE) to contact football has also been associated with neurological and psychiatric dysfunction in later life (23, 34, 44). Childhood and adolescence is a crucial period of immense neurological development and maturation (45). More specifically, ages 9–12 have been identified as key periods corresponding to peak gray and white matter volumes and neurological maturation of the hippocampus and amygdala (45, 46). The vulnerability of the brain during this critical period may be exploited through brain trauma via tackle football, although others argue that the neuroplasticity of the brain may compensate for the behavioral dysfunction from repeated trauma (28, 47). Alosco et al. (23) demonstrated that AFE<12 was associated with more than double the risk of cognitive impairment in later life. This was characterized by impairment in correlates of apathy (OR, 95% CI: 0.86, 0.76–0.97) and depression (OR, 95% CI: 0.85, 0.74–0.97), but not cognition (23). Schultz et al. (16) has shown similar findings, suggesting AFE to lead to detrimental changes in brain architecture in later life. In 86 symptomatic former NFL players, decreased thalamic volume was correlated with number of years played [*p* = 0.03 (left hemisphere), *p* = 0.03 (right hemisphere)]. Additionally, younger AFE has also been related to reduced left thalamic volume (*p* = 0.014) (16), and alterations in corpus callosum architecture (44), further highlighting the detrimental effects of repetitive head impacts.

## Epidemiology of CTE

Quantifying the epidemiology of CTE requires further elucidation, as studies have largely involved cohorts with a history of repetitive brain trauma such as athletes (boxers, football players, wrestlers). This has led to an overestimation of prevalence (5) due to the incorporation of biased sampling methods such as lack of a control group. This is further exacerbated by the incapability to definitively diagnose CTE during life (31, 48). However, the studies that aim to identify the prevalence of CTE in football players have shown unequivocal findings. In the largest ever case series of CTE, involving 202 deceased former football players, Mez et al. (49) demonstrated the existence of CTE in 87%, including 99% ex-NFL Players (49). Additionally, the magnitude of disease burden was correlated to level of play, with high school athletes having mild CTE, and NFL players showcasing the most severe form of CTE. In a retrospective cohort study of 3,493 NFL players, Lehman et al. (50) concluded the risk of mortality due to neurodegenerative causes to be 3x higher than the general US population, and 4x higher for ALS and AD (50). Additionally, ex-NFL players over the age of 50 have also been shown to be 5x more likely to be diagnosed with dementia than national population averages (51).

A history of brain trauma also appears to be an antecedent for the onset of CTE. In a sample of 1721 cases of contact sport athletes, (52) reported CTE in 32% of cases, whereas no cases of CTE were found in patients with no prior brain trauma in the control group (52). This suggests that contact sport is a predisposing risk factor in the development of CTE. While the exact incidence of CTE is unknown, it may be attributed to duration of play, position, sport, age of first exposure, and genetics (32). To better quantify the prevalence of CTE, prospective studies are required to compare individuals with a history of repetitive brain trauma to healthy controls.

## Diagnosis of CTE

Although emerging evidence has highlighted the use of 2-(1-{6-[(2-[F-18] fluoroethyl) (methyl)amino]-2-naphthyl}ethylidene) malononitrile-positron emission tomography ([F-18] FDDNP PET) and other neuroimaging techniques for a preliminary pre-mortem diagnosis (53, 54), a definitive CTE diagnosis is confirmed with post-mortem autopsy and immunohistochemistry for p-tau (15, 19, 26, 55). Generally, an autopsy of CTE will show distinct features that distinguish it from other taupathies such as Alzheimer's Disease (AD). This has offered some clarity in regard to the pathophysiological differences between the two diseases. The National Institute of Neurological Disorders and Stroke/National Institute of Biomedical Imaging and Engineering (NINDS/NIBIB) consensus panel recently identified key preliminary findings of CTE (34, 56) and concluded that certain distinguishing features were sufficient to confirm a CTE diagnosis based on macroscopic and microscopic abnormalities. McKee et al. (34) also proposed a 4-stage classification system for CTE based on the extent of lesions and disease severity (See Table 1) (34). Prior to this, Omalu and colleagues proposed 4 distinct phenotypes of CTE (57).

**Table 1:** Grading system of CTE, based on a provisional four-stage classification scheme centered around the extent and severity of tau pathology.

**Stage**	**Criteria**
I	1 or 2 focal perivascular CTE lesions in cerebral cortex at the depths of the sulci.
II	>3 CTE lesions in multiple cortical regions and superficial NFTs along the sulcal wall and gyral crests.
III	Multiple CTE lesions, widespread cortical NFTs, and NFTs in the medial temporal lobes.
IV	Multiple CTE lesions, widespread cortical NFTs, NFTs in the medial temporal lobes, widespread astrocytic p-tau pathology, neuronal loss, and gliosis.

*Note: Aß amyloid plaques are entirely age-dependent and have no relationship to staging. Adapted from (56) with permission*.

## Macroscopic and microscopic findings in CTE

The classical signs of CTE induced macroscopic changes are more commonly found in advanced forms of CTE, and rarely manifest in early stages (26). They include cerebral atrophy, reduced brain mass, enlarged lateral and third ventricles, cavum septum pellucidum, pale locus coeruleus, and diencephalon atrophy (19, 26, 34, 56). The required criteria for a diagnosis of CTE encompasses aggregates of hyperphosphorylated tau (p-tau) in glial cells such as astrocytes, neurons, and cell processes, within the vicinity of small vasculature and in the cortical sulci, which typically presents in an irregular pattern (34, 45). CTE and AD share have many similarities, as well as distinct differences (58). The primary criteria for the diagnosis of AD involves the post-mortem finding of Aβ amyloid and neuritic amyloid plaques in a laminar distribution, in the middle frontal gyrus, the superior and middle temporal gyri, and the inferior parietal lobule (59). Secondary amyloid depositions may also be found in areas such as the cerebellum and basal ganglia. These findings contrast with those in CTE cases, where Aβ plaques may be found in a diffuse pattern in sporadic loci, or none at all. In the case of AD, the location p-tau NFTs should largely overlap with those of Aβ amyloid (59), as well as their presence in the hippocampus (60). This contrasts with CTE, where definitively, p-tau aggregates are classically found in a spot-like fashion in the perivascular spaces around blood vessels, deep in the cortical sulci (34, 56). Supportive findings also include NFTs in the superficial layer (II-II) of the cerebral cortex, in cortical area 2 and 4 of the hippocampus. Additionally, the dimensions of NFTs in CTE are usually larger in size than those seen in AD (34, 61). Transactive response DNA-binding protein 43 (TDP-43) aggregates are also a common finding in the vast majority CTE cases, co-localized with p-tau NFTs (56, 58, 62). Although TDP-43 aggregates are found in AD cases, they are not co-localized with NFTs (63), which is another point of distinction between AD and CTE. Neuropathological sampling of TDP-43 should first be taken from the amygdala and hippocampus, and if positive, should also be investigated for in the temporal pole and frontal cortex (34, 56). Owing to the irregularities in lesion distribution however, additional sampling may also be required based on Alzheimer Disease Centers (National Institute on Aging-Alzheimer's Association (NIA-AA) criteria (64).

## Symptoms and cognitive function

Typically, symptoms of CTE do not present immediately and typically occur in midlife, usually decades after the initial insult. In 51 confirmed cases of CTE, the mean onset of symptom presentation was 42.8 years of age (*SD* = 12.7) after initial exposure, but ranges from 25 to 76 years of age (14). Stein et al. (19) compiled confirmed cases of CTE from two studies and concluded an average of 15 years before symptom presentation, while the mean age of death was shown to be 59.3 years of age (14, 19). On average, symptom onset usually occurs around 8 years after retirement (14, 32), with a small proportion (33%) of athletes symptomatic at the time of retirement, and 50% becoming symptomatic within 4 years post-retirement (14). It is unclear why this latency period exists, but is believed to be a result of tau propagation from focal to widespread areas, as a consequence of progressive axonal disruption (4, 15). The symptoms of CTE typically present in sequelae of irritability, aggression, episodic memory impairment, cognitive dysfunction, suicidal ideation, rampant mood fluctuations, and depression (10, 14, 32, 49). This early symptomatic period is also associated with substance abuse, and high frequency of suicide (14, 57). As the disease progresses, more severe symptoms begin to manifest such as altered motor function and parkinsonian symptoms (3, 65). In its most advanced form, CTE may be difficult to distinguish from high-grade AD (3, 15, 57). However, owing to individual variability, not all patients will present with a latency period between brain trauma and symptomology (57). Indeed, Stern et al. (67) report CTE to present in two distinct patterns:

1. a young-age onset with initial behavior/mood perturbations that manifests at around age 35. These include impulsivity, aggressiveness, violent tendencies, and progression to deficits in cognition.
2. Late-age onset with cognitive deterioration, which presents around age 60. This subset is characterized by deficits in executive function and attention memory. This phenotype typically displays features of advanced CTE (66, 67). Individuals may also manifest symptoms via alterations in behavior and mood.

Previous work has also shown CTE to synergistically exist with other neurological disorders, such as Lewy Body Dementia (LBD), motor neuron disease such as Amyotrophic Lateral Sclerosis (26, 68) and AD (15, 26, 39, 52, 69). Dickstein et al. (70) has shown that 37% of ex NFL players with CTE meet diagnostic criteria for other neurodegenerative disorders such as LBD (70). This highlights the fact that the disease may not exist independently in its pure form (71) and may be attributed to widespread axonal disruption and progressively increased p-tau hyperphosphorylation, which serves as a stimulus for the deposition of other proteins such as β-amyloid, αβ-synuclein, and TDP-43 (3, 15). However, due to a lack of high-quality longitudinal studies, questions remain in regard to precise symptomology, warranting more research.

## CTE as a risk factor for suicide and mortality

Despite prevailing media reports of several ex-NFL players taking their own lives, and post-mortem showing hallmark traits of CTE, no causal link exists between CTE and suicide (5). Previous work has shown that retired NFL players have a statistically lower risk of suicide compared to the general American population (72, 73). Despite the increased neurodegenerative mortality of this population (50), Lehman et al. (73) concluded that professional football players do not have an increased risk of suicide, but rather have a lower standard mortality rate (SMR) than the general USA population (SMR = 0.47;95% CI:0.24–0.82) (50, 73). Others have shown similar findings in NFL players, compared to race/ethnicity matched general population (72, 74), likely related to the healthy worker effect (74). A recent study has shown that playing in the NFL was not associated with an increased risk of long-term all-cause mortality (75). A leading cause of death of NFL players has been shown to be cardiometabolic disease (75), which may be attributable to increased body-mass index in specific positions, such as defensive linemen (74). Others have shown neurodegenerative causes to be the main cause of death, due to evidence of other co-morbidities such as LBD and Parkinsonian-related causes (49). The belief that CTE predisposes American football players to suicide is rooted in several high-profile cases (76) since 2009, which has led to an overestimation of suicide prevalence based on selection-bias (33). A retrospective cohort study examined mortality from suicide between 1920 and 2015 in a sample of 26,702 professional American football players and reported 26 suicides during this time period. 43% of these cases happened after 2009 and involved high profile players (77) such as Junior Seau, a hall-of-fame linebacker who ended his life, and was later diagnosed with CTE post-mortem (76, 78).

## Future directions in potential *In-vivo* diagnostics

Although studies of CTE have involved post-mortem analyses, [F-18] FDDNP PET is a promising tau imaging ligand that could be used 1 day to detect brain trauma *in-vivo*. In professional football players, [F-18] FDDNP PET has been shown to be an archetypal tau ligand, as the imaging pattern seen presents in a characteristic topographical distribution consistent with that of CTE (79), such as increased signal intensity in the medial temporal and frontal lobes (53). Additionally, [F-18] FDDNP PET binds preferentially to NFTs, p-tau, and β-amyloid (80). However, the few studies that exist on this imaging modality are limited by sample size. As such, larger trials are necessary to determine the true sensitivity and specificity of [F-18] FDDNP PET. PET imaging has been purported to be in its nascent stage in CTE diagnosis (80), however, significant strides are being made.

Another emerging imaging modality for consideration in this context is magnetic resonance imaging (MRI). The safety profile and consideration of magnetic resonance spectroscopy (MRS) as a “virtual biopsy” are conducive to inclusion in the emerging search for tools to diagnose CTE (81). Neurometabolites such as N-acetyl aspartate, choline, and glutamine/glutamate (NAA, Cho and Glx) are shown to have similar changes on MRS imaging to CTE pathological changes, although not specific (81). More recently, Ruprecht et al. (82) included 25 studies for qualitative synthesis in their systematic review on the characterization of CTE by MRI and MRS, finding that there is only partial comparability amongst published studies given presentation of diffusion MRI, structural MRI, functional MRI, and MRS (82). These authors found gender bias and inconsistently applied age-matched controls limit direct comparison. In general, current limitations of CTE assessment with MRI are studies with small sample sizes, lack of disease comparison groups, and inability to confirm CTE diagnosis *in*-*vivo*, rendering elucidation of a direct relationship between MRS and CTE currently lacking. Further ante- and postmortem research in the use of MRS is acknowledged to be necessary (81, 82).

Recently, the field has also advanced in identifying potential biomarkers of traumatic brain injury. There are a range of biomarkers approaching clinical validation (glial fibrillary acidic protein, S100B) and more emerging, such as microtubule associated protein-2 (MAP-2) and brain-derived neurotrophic factor (BDNF) (83, 84). In the future, the use of biomarkers in conjunction with diagnostic imaging, such as PET and MRI, offers a promising avenue for early diagnosis, monitoring of progression, and timely therapeutic interventions.

## Future directions in potential therapeutics

Recent work has shed light into the molecular etiopathogenesis of CTE, specifically the inflammatory cascade that results in hyperphosphorylated p-tau accumulation. A seminal paper by Lucke-Wold et al. (30), has shown that post-translational modification involves ac-tau at the lysine 280/281 (K280/K281) position in the paired helical filament-6 (PHF-6) motif, prior to hyperphosphorylation of the tau protein (30). Acetylation has been purported to be the initiating event that results in the vicious cycle of repetitive damage and imperfect repair, which may be from increased acetyltransferase enzyme p-300 levels, shifting the enzyme milieu to favor kinases over phosphatases (85). This “two-hit” mechanism of acetylation and phosphorylation leading to p-tau aggregation via microtubule destabilization provides the framework for future studies to inhibit this pathway. However, efforts aimed at inhibiting tau phosphorylation have elicited minimal advances in treatment. A new strategy could perhaps be pharmacological therapies aimed at inhibiting ac-tau in PHF-6. Studies in murine models have shown that salsalate, a non-steroidal anti-inflammatory drug (NSAID) reduces p-300 mediated acetylation of tau (30), resulting in improved cognition and reduced atrophy of brain regions such as the hippocampus. Lagraoui et al. (86) reported similar findings in a murine model of traumatic brain injury (TBI) (86). Salsalate was shown to inhibit inflammatory pathways after TBI proximal to the injury site via reduction in microglia/macrophage activation. Additionally, salsalate was also shown to induce genes implicated in neurogenesis (such as Tbr2) and neuroprotection (oxytocin and thyrotropin releasing hormone), while also improving functional recovery post-injury (30, 86). The earliest reported proof of principle study demonstrating that p-300 inhibition could be used to treat tauopathies was demonstrated in a seminal paper by Min et al. (87). Others have recently identified that methylene blue (MB), an inhibitor of tau aggregation, also modulates acetylation activity at K280/K281 (86), and may thus be promising if findings are consistent in large-scale human clinical trials. This may be explored in the future, in conjunction with therapies that enhance deacetylase activity such as HDAC-6 and sirtuin-1 (88) for synergism and potentiation. It is also crucial to prevent other steps involved in tau propagation such as tau oligomerization and dissemination from focal to widespread areas. Considering that hyperphosphorylated tau spreads in a prion-like manner, preventing this spread through p-tau monoclonal antibodies and competitive inhibitors of prion protein receptors that facilitate tau oligomer toxicity is also an area of consideration for future work (89). These new studies highlight the potential role of pharmacological therapy. In order for the field to move forward in terms of therapeutics, the development of drug targets should be multipronged as to target different stages of the inflammatory cascade after brain injury.

**Table 2:** Typical microscopic and macroscopic features found in each stage of CTE.

**Stage**	**Typical Macroscopic appearance**	**Typical Microscopic appearance**
I	Unremarkable gross appearance and weight Occasional enlargement of frontal horns of the lateral ventricles	One of two, Isolated perivascular foci of p-tau NFT and neurites, the neurites are often dotlike; the perivascular foci are commonly found at the depths of the sulcus of the superior and dorsolateral frontal and temporal cortices p-tau NFTs may be found in the locus coeruleus
II	Unremarkable appearance and weight Pallor of the locus coeruleus and substantia nigra Enlargement of the frontal horns of the lateral ventricles	Multiple (> 3) perivascular foci of p-tau NFTs in the frontal, temporal or parietal cortices NFTs in the superficial cortical layers of cortex, especially in the temporal lobe p-tau NFTs in the locus coeruleus, substantia nigra and substantia innominata Sparse TDP-43 positivity in medial temporal lobe
III	Mildly reduced brain weight, atrophy in the frontal and temporal lobes Enlargement of the third and lateral ventricles Septal abnormalities, ranging from mild septal perforation to complete atrophy Atrophy of the thalamus, hypothalamus, posterior corpus callosum and mammillary bodies depigmentation of the locus coeruleus and substantia nigra	NFT pathology in widespread cortical regions NFTs in the locus coeruleus, substantia nigra, subs innominata Diffuse NFTs and neurites in hippocampus, amygdala, and entorhinal cortex Sparse NFT in the thalamus, nucleus accumbens TDP-43 positivity in the cerebral cortex, and medial temporal lobe
IV	Significantly reduced brain weight Extensive atrophy throughout the brain, including white matter Significant enlargement of the third and lateral ventricles Marked septal abnormalities, as in stage III Severe pallor of the locus coeruleus and substantia nigra in all cases	Diffuse white matter rarefaction Dense p-tau NFT and glial pathology distributed throughout the brain (cerebrum, diencephalon, basal ganglia, and brainstem) and spinal cord Marked NFT in medial temporal lobe structures NFT in LC, SN, SI NFT in basis pontis, mammillary bodies, dentate nucleus of cerebellum TDP-43 inclusion bodies and neurites in cortex, medial temporal lobe, brainstem

*Note: This is not the criteria used for making the staging designation (refer to Table 1). Adapted from (56) with permission*.

## Current state and future directions

What is most concerning about NFL-associated CTE cases is their preventable nature. There is a preponderance of ex-NFL players that allege the NFL failed to provide information regarding the neuropsychiatric risks associated with playing professional football (78, 90). Recently, the NFL implemented numerous safeguards following increased awareness of CTE. These include limiting contact practices involving tackles to 1x/week and penalization of direct head impacts with substantial fines and disciplinary action. Additionally, an NFL associated website (https://www.playsmartplaysafe.com/) highlights the leagues updated concussion protocols and return-to-play guidelines; the NFL works to ensure concussed players adhere strictly to these return-to-play guidelines after brain trauma. This is in an effort to prevent exacerbation of injury, or occurrence of a second episode (91). Non-compliance with established concussion protocol is a current issue. This process can be long and there is an incentive to recover as quickly as possible (92). The result is an under-reporting of symptoms by athletes, to ensure a quicker return to play (93). Athletes and others involved in professional sports do not always consider the long-term implications that contact sports and repetitive head injury can have on their neuropsychiatric functioning in the future, and that is why we believe changes must be made.

## Conclusions

Our understanding of CTE in American football players has improved immensely since the first case reports of Omalu and colleagues. It has now emerged that CTE has afflicted football players from bouts of head trauma. Despite these advances, more work is necessary to elucidate the underlying pathophysiological mechanisms, and the incidence and prevalence of disease burden in the general populace. There is a need to move beyond case reports and focus on more systematic studies examining CTE in sport and non-sport populations. At present, there is also no widely accepted clinical criteria for a diagnosis of CTE despite the preliminary work of McKee et al. (56) and others (94, 95). The field is moving forward with biomarkers nearing appropriate validation and *in-vivo* diagnostic techniques such as MRI and [F-18] FDDNP PET being explored, and therapeutic modalities showing promise in murine models. However more clinical human studies are warranted for timely diagnosis and appropriate management of CTE.

## Author contributions

TT was responsible for conception of the mini-review, manuscript drafting, submission, and was involved in all aspects of this paper. MI assisted in manuscript preparation and wrote pathological findings section. IT provided advice and guidance during the completion of the manuscript and assisted in submission process. PG-R wrote the future directions in *in vivo* potential diagnostics section. TCT assisted in manuscript drafting and wrote the future directions section. TAT critically edited the manuscript for intellectual content, and provided guidance. All authors edited and approved the final version of the manuscript, and agree to be accountable for all aspects of this work. The authors have no conflicts of interests to declare.

### Conflict of interest statement

The authors declare that the research was conducted in the absence of any commercial or financial relationships that could be construed as a potential conflict of interest.
